# Salmonella enterica subsp. *arizonae* Isolated from a Canine Clinical Case of Prostatitis

**DOI:** 10.1128/MRA.00118-20

**Published:** 2020-03-26

**Authors:** Mary L. Krath, Sara V. Little, Andrew E. Hillhouse, Sara D. Lawhon

**Affiliations:** a Department of Veterinary Pathobiology, College of Veterinary Medicine & Biomedical Sciences, Texas A&M University, College Station, Texas, USA; b Texas A&M Institute for Genome Sciences and Society, College of Veterinary Medicine & Biomedical Sciences, Texas A&M University, College Station, Texas, USA; Loyola University Chicago

## Abstract

This is an announcement for the genome sequence of a clinical isolate of Salmonella enterica subsp. *arizonae* isolated from the urine and prostate of a 6-year-old male Labrador retriever. This is one of the few reports of a Salmonella enterica subsp. *arizonae* isolate cultured from canine urine.

## ANNOUNCEMENT

Salmonella enterica subsp. *arizonae* was originally isolated from a reptile in Tucson, Arizona, in 1939 and has since been recovered from a variety of animals, particularly reptiles, from around the world (1–10). This organism rarely causes disease in reptiles but is well recognized as a cause of infection in humans, particularly in children (11–19). Infections may be life-threatening and can include gastroenteritis, osteomyelitis, pneumonia, and meningitis (12, 14–17). The isolate sequenced here was isolated from the urine and a prostate wash from a 6-year-old, castrated male Labrador retriever. Cultures of the prostate continued to be positive for *Salmonella* for 3 months following initial isolation, but subsequent cultures at 4 and 5 months were negative, and the patient was considered cured. The patient was also positive for Dirofilaria immitis, *Ehrlichia* species, and Anaplasma phagocytophilum and was treated for these infections. *Salmonella* is rarely cultured from canine urine, but a case in a dog undergoing immunosuppression to treat immune-mediated polyarthritis was reported in 2018 (20).

Patient specimens were used to inoculate Trypticase soy agar supplemented with 5% sheep’s blood (blood agar plate [BAP]). Cultures were incubated at 35°C ± 2°C in an atmosphere supplemented with 5% CO_2_. The organism was identified using matrix-assisted laser desorption ionization–time of flight (MALDI-TOF) mass spectrometry (Bruker Biotyper). The identification was confirmed using PCR as previously described (21). The organism was submitted to the National Veterinary Services Laboratory for serotyping and determined to be S. enterica subsp. *arizonae* serotype 41:z4,z23:−. Isolates were stored at −80°C in brucella broth supplemented with 10% glycerol and were revived for sequencing by inoculating an aliquot of the frozen bacteria onto a BAP.

An isolated colony was used to inoculate lysogeny broth (LB) at 37°C with shaking. A 1-ml aliquot was then taken and lysed using Macherey-Nagel type B bead tubes and NucleoMag lysis buffer in a Qiagen tissue lyser machine. Genomic DNA was extracted according to the manufacturer’s instructions (Macherey-Nagel). The quality of the DNA was determined using an Agilent genomic DNA TapeStation run.

Default settings were used for the following steps unless specified otherwise. The Illumina Nextera DNA Flex library preparation kit and the Illumina MiSeq V3 2 × 300 kit were used for library preparation and sequencing. The data from the run were uploaded to Illumina BaseSpace for quality control and FASTQ generation. The genome was sequenced with paired ends, resulting in a draft genome of 3,020,960 reads of 251 bp with 165× coverage.

SPAdes version 3.13.0 was used for assembly with the careful parameter (22). The assembly was composed of 145 contigs. The assembled genome was 4,623,155 bp long. The genome’s GC content was approximately 51%. The Benchmarking Universal Single-Copy Orthologs (BUSCO) score was 99% based on the *Enterobacteriales* OrthoDB version 9 data set (23, 24). The genome was annotated using the NCBI Prokaryotic Genome Annotation Pipeline (PGAP) version 4.11 (25, 26).

### Data availability.

This genome sequence was submitted to GenBank under the accession number JAAAGD000000000. The raw reads have been submitted to the SRA and can be found under accession number SRR10876209. Both the genome sequence and raw reads are under BioProject PRJNA600881.
